# Acceptance and commitment therapy- based intervention to improve psychological skills and resilience in surgical trainees: a randomised waitlist-controlled trial

**DOI:** 10.1186/s12893-025-03059-5

**Published:** 2025-07-28

**Authors:** Maddy Greville-Harris, Agata Wezyk, Kevin Thomas, Stephen Richer, Helen Bolderston, Nyssa Purchase, Sine McDougall, Kevin J. Turner

**Affiliations:** 1 Department of Psychology, Poole House, Bournemouth University, Fern Barrow, Poole, BH12 5BB UK; 2 Department of Urology, University Hospitals Dorset, Royal Bournemouth and Christchurch Hospitals NHS Foundation Trust, Castle Lane East, Bournemouth, BH7 7DW UK

**Keywords:** Surgeon wellbeing, Acceptance and commitment therapy, ACT, Wellbeing intervention

## Abstract

**Background:**

High levels of burnout, psychological distress and suicidal ideation are well documented in surgeons. The need for supporting wellbeing of surgical trainees and preparing them for the inevitable occupational stress have also been identified in the literature. ACT-based interventions have been successfully utilised in other populations to help develop psychological skills and improve wellbeing. However, there has been no research focusing on such interventions for surgical trainees. Therefore, this randomised controlled trial (RCT) examined the impact of an Acceptance and Commitment Therapy (ACT) based intervention on key psychological skills and wellbeing outcomes in surgical trainees.

**Methods:**

Surgical trainees (*n* = 68) were randomised to a three-session ACT-based intervention or waitlist control group. Validated scales were used to measure change in psychological skills (values consistency, psychological flexibility) and wellbeing outcomes (resilience, self-compassion and emotional distress) pre, during and post intervention, and at 3-month follow-up.

**Results:**

Two-factor mixed analyses of variance (ANOVAs) with post hoc pairwise comparisons indicated significant improvements in values consistency (*p* <.001), resilience (*p* <.001) and self-compassion (*p* <.001) for the ACT-based intervention compared with controls from baseline to follow-up. No such improvements were observed for psychological flexibility or emotional distress.

**Conclusions:**

This RCT suggests that a short ACT-based intervention is useful for surgical trainees, showing promise in facilitating improvements in values consistency, resilience and self-compassion. Future research is needed to explore the scalability of such interventions, as well as the potential need for more tailored mindfulness training within such trainings, to specifically target psychological flexibility and reduce emotional distress.

**Trial registration:**

Preregistered with CLINICALTRIALS.gov Protocol Registration and Results System, NCT03759795, first posted 30^th^ November 2018.

**Supplementary Information:**

The online version contains supplementary material available at 10.1186/s12893-025-03059-5.

## Background

The impact of occupational stress on surgeons is well-documented; stress-related harm in surgeons includes burnout [1], depression and anxiety [2], substance abuse [3], suicidal ideation [4], and impaired performance [5]. The extent of this harm is related to the degree and chronicity of the stress [6, 7], with adverse events in surgery recognised as a particularly potent stressor [8]. Indeed, post-traumatic stress symptomatology after adverse events is akin to that experienced by military personnel returning from conflict [9].

Arguably there is a need to prepare trainee surgeons with the skills to manage such stress. Yet the profession has struggled to determine how to go about this; in a recent study 72% of trainees reported being personally affected by a patient’s death, nearly a third reported secondary trauma, with over half reporting they did not feel adequately supported to deal with such emotional experiences [10]. Indeed, it has been suggested that “perhaps no other profession that demands elite-level physical performance has devoted so little to the well-being of its practitioners, much less its trainees” (p. 1017) [11].

Given the well-being challenges surgeons experience at work [12], it is important for surgical trainees to learn psychological skills to cope with these emotional demands. Incorporating suitable initiatives into medical training can improve the well-being of trainee surgeons, as well as improving patient care [13], and potentially lengthening surgical careers [14].

One promising approach to psychological interventions for surgical trainees might be Acceptance and Commitment Therapy (ACT), which aims to promote psychological flexibility. This involves accepting the present moment without judgement, noticing the inevitable distressing thoughts, emotions, or sensations, that arise without becoming ‘hooked’ by them [15]. Thus, one can act in alignment with personal values in a meaningful way (value consistency) despite the presence of difficult thoughts and feelings which may otherwise serve as barriers to such action [16]. There is little work examining the value of building psychological flexibility skills in surgeons; however, a recent study found that psychological flexibility played a role in predicting surgeon well-being [17].

ACT-based trainings focusing specifically on developing psychological flexibility (through mindfulness and value-based work), have been found to be effective across a range of work settings (e.g [18, 19]). Such interventions have been found to decrease trauma-related symptoms [20], improve mental health [21], psychological distress [19], and self-compassion [22]. The latter, defined as holding non-judgmental stance towards personal suffering along with the desire to understand and alleviate it [23], may be particularly useful in protecting against burnout and secondary trauma in surgeons [24].

More broadly, ACT-based interventions have been found to: (1) foster self-compassion, mindfulness, and psychological flexibility and, (2) reduce general psychological distress, work stress, and burnout in healthcare professionals [25–27]. One study [18] evaluated the effectiveness of a one-day ACT-based workshop for healthcare workers experiencing distress. Participants in the intervention group reported lower psychological distress three months post-intervention than those in the waitlist group. Similarly, a randomised controlled trial (RCT) [19] with healthcare staff (*n* = 98) compared the impact of a four-session ACT intervention versus waitlist control. The intervention led to improvements in psychological distress, with nearly half of the ACT group showing clinically significant change in their symptoms.

While recent studies have explored the usefulness of mindfulness-based interventions more generally for surgical trainees and found promising outcomes in terms of reduced burnout [28] and stress [29], no studies have explored the usefulness of a purely ACT-based intervention. The present study will test an RCT, hypothesising that scores on measures of the psychological skills (psychological flexibility, value consistency) and wellbeing outcomes (self-compassion, resilience, and emotional distress) will improve for surgical trainees exposed to an ACT-based intervention relative to a waitlist control (WLC) group.

## Method

### Design

A parallel group trial RCT was implemented comprising a two-factor mixed within- and between-groups design. This compared the effectiveness of a three-session ACT intervention with waitlist control (WLC). The between-groups factor (training intervention) comprised two levels: ACT versus WLC. The within-groups factor (time point) comprised five levels: baseline, 1-week post session 1 (T1), 1-week post session 2 (T2), 1-week post session 3 (T3), and 3-month Follow-up.

### Participants

Trainee surgeons (all Speciality Registrars, StR 1–8) were recruited from 8 NHS Trusts in Southern England using opportunistic sampling. Senior surgeons advertised the study to trainees in their units. Participants were not offered reimbursement for taking part, but those in the WLC were offered the ACT intervention after completing the RCT, to ensure that all participants in the study had the option to utilise the ACT resources.

An a priori power analysis was used to estimate the sample size needed to detect a medium effect. The medium effect size estimate was in line with a recent mindfulness-based intervention for surgeons targeting emotional exhaustion [28] and comparable effect sizes seen for perceived stress in ACT-based intervention trials for other health professionals [30, 31]. With the parameters set at alpha = 0.05 and power = 0.8, a total sample size of 50 or more participants was estimated as sufficient to detect a medium effect size (Cohen’s d = 0.5).

### Outcome measures

The questionnaire for this study comprised demographic questions (e.g. age, gender, ethnicity, specialty) followed by a series of validated scales, as outlined below. The Cronbach’s alphas (α) for scales at each timepoint among our sample are outlined in Additional Files 1.

#### The Brief Resilience Scale (BRS)

The BRS [32] comprises six items (e.g., *I tend to bounce back quickly after hard times*), each measured on a 5-point Likert scale where higher scores denote higher resilience. The instrument is a reliable and widely used unidimensional scale [33, 34], with Cronbach’s alphas typically ranging from 0.80 to 0.91 [35]. Post-collection but pre-analysis, the data for item 1 on the 6-item BRS were corrupted and regarded as insufficiently reliable for analysis. Consequently, items 2–6 on the scale were summed to form a 5-item scale (BRS-5) for each timepoint (score range 4–20). This reduced scale had acceptable internal reliability (see Additional Files 1).

#### The Self-Compassion Scale (SCS)

The SCS [36] comprises 26 items (e.g., *I’m tolerant of my own flaws and inadequacies*), each measured on a 5-point Likert scale. A total score was computed after reverse coding negative subscale items, with higher sum scores denoting higher self-compassion (score range 26–130). The instrument has good reliability [36], with Cronbach’s alphas typically ranging from 0.75 to 0.81.

#### Acceptance and Action Questionnaire- II (AAQ-II)

The AAQ-II [37] is a unidimensional scale measuring general psychological inflexibility. It comprises 7-items (e.g., M*y painful memories prevent me from having a fulfilling life*), each measured on a 7-point Likert scale. The scale has good reliability and internal consistency (mean α = 0.88) [37]. A sum score was calculated with higher scores denoting higher psychological inflexibility (score range 7–49).

#### The Work-related Acceptance and Action Questionnaire (WAAQ)

The WAAQ [38] measures psychological flexibility in the context of workplace functioning. It comprises 7 items (e.g., *I am able to work effectively in spite of any personal worries that I have*), each measured on a 7-point Likert scale. Higher scores indicate higher psychological flexibility (score range 7–49). The WAAQ has demonstrated good reliability [39], (e.g. α = 0.85). We used both the WAAQ and AAQ-II measures, because whilst the AAQ-II is the most widely used measure of general psychological flexibility [38], it does not capture context specific aspects of work-related psychological flexibility. These two measures have been found to capture different aspects of psychological flexibility; the AAQ-II is associated with life satisfaction/flourishing, whereas the WAAQ relates to aspects of psychological flexibility related to absorption with work engagement [40].

#### The Valued Living Questionnaire II, Composite Scale Measure (VLQC)

The VLQC [41] comprises two subscales. The first subscale measures the importance of 10 life domains (e.g., family, parenting, education/training, physical self-care) using a 10-point Likert scale per item. The second subscale focuses on how consistently respondents have lived in accordance with each of the 10 life domains, each measured on a 10-point Likert scale. The composite score is calculated by multiplying scores on the two subscales for each domain and then calculating a mean score. Composite scores range from 10 to 100 and give a measure of how consistently respondents acted in accordance with important values. Due to the composition of the scale (examining independent domains), a Cronbach alpha statistic is not a useful indicator of reliability and so was not calculated.

#### The Depression Anxiety Stress Scales (DASS-21)

The DASS-21 [42] measures negative emotion states of depression, anxiety and stress. The scale comprises 21 items across three 7-item subscales: depression (e.g., *I felt down-hearted and blue*), anxiety (e.g., *I felt I was close to panic*) and stress (e.g., *I find it hard to wind down*). The items are measured on a 4-point Likert scale where higher scores denote higher stress, depression, and anxiety (total score range = 0–63). The instrument has good reliability, with Cronbach’s alphas ranging from 0.81 to 0.88 for each of the subscales [43]. In line with previous work, this study uses the composite measure as an indicator of general levels of distress [44].

### Intervention

The ACT training was delivered by one of the research team following a well-established protocol, based on the intervention set out by Flaxman et al. [45]. The intervention comprised three sessions: (1) Welcome and Introduction to ACT-based training; (2) Untangling from Internal Barriers to Value-Based Action; (3) Consolidation of Mindfulness and Value-Based Action Skills. The protocol was delivered by researcher SR under the supervision of HB (Clinical Psychologist, experienced ACT therapist and trainer). Prior to delivering the training, SR attended a 4-day ACT training but had no previous experience of ACT or facilitating psychological interventions. SR familiarised himself with the ACT-informed protocol outlined by Flaxman and colleagues [45] and then attended supervision with HB approximately every two weeks during the intervention delivery. The structured and manualised approach used allowed for training to be delivered by a non-therapist with little prior experience of the full ACT therapy model.

**Fig. 1:**
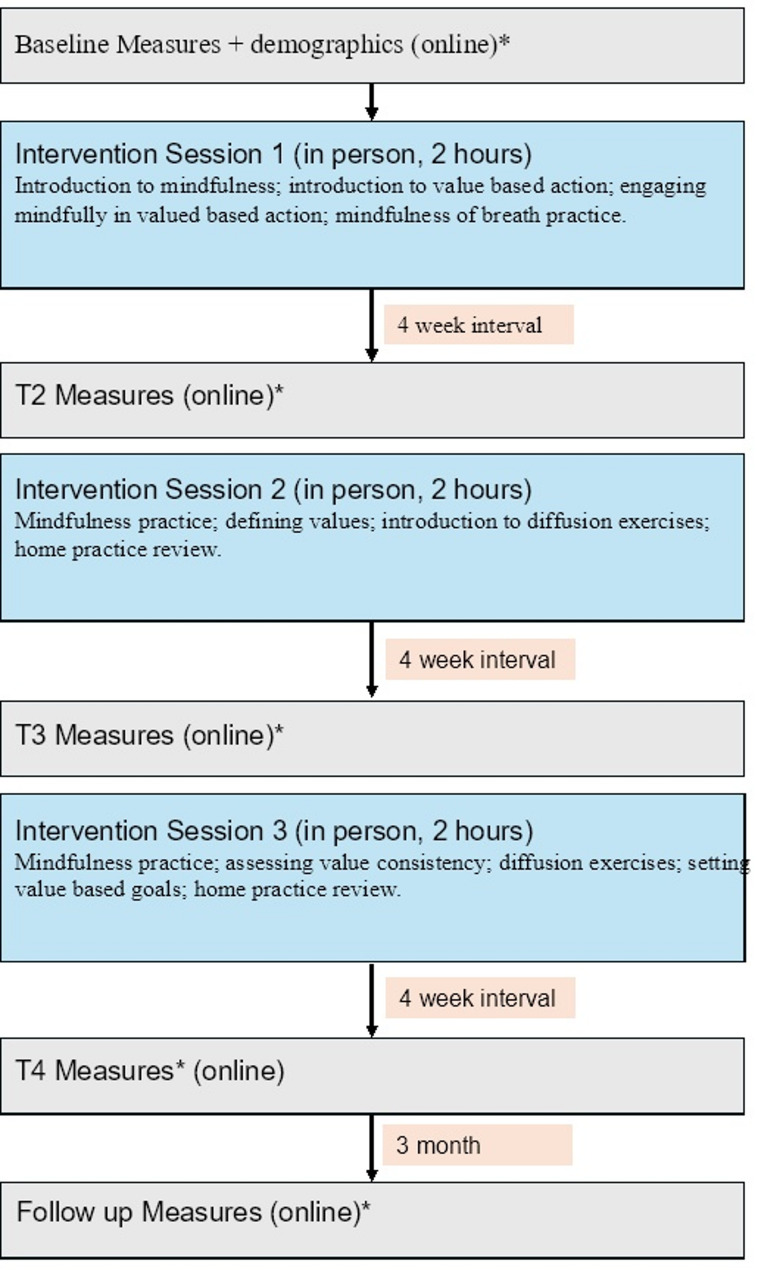
Flow diagram of content/timings of data collection and intervention sessions

The training was delivered one session every 4 weeks, following the format outlined in Fig. 1. Flaxman and colleagues [45] suggest for this manualised ACT training to be carried out weekly but acknowledge the need for adjustment to suit the practical restraints of the sample. Given the busy work schedule of trainee surgeons, we offered sessions 4 weeks apart to allow time for participants to practice their mindfulness skills and implement value-based goals in between sessions.

Based on evidence that a brief three-session ACT intervention is effective in facilitating change in psychological outcomes in occupational settings [45], our intervention comprised three 2-hour sessions. Each session comprised: brief mindfulness practice; review of homework set in the previous session (in sessions 2 and 3); training in ACT-based skills; teaching in the roots of ACT; mindful review of the session; and setting of home practices (see summary of content in Additional Materials 2).

Study recruitment started in January 2018 with intervention delivery continuing until May 2020. Each session included home practice exercises, which provided an opportunity for participants to use the skills and techniques introduced during the sessions and practice these during the month interval between sessions. Although the ACT protocol was initially intended for group sessions, this study utilised one-to-one sessions due to: (1) the practical difficulties of finding an appropriate timeslot when all participants were free to attend, (2) informal conversations with surgeons who indicated that they would be less likely to attend a group-based intervention, and: (3) the potential usefulness of individual sessions to encourage personal reflections by participants. Participants thus received the intervention individually, with most sessions (*n* = 81; 84%) being delivered in-person. However, 15 session 3 interventions had to be delivered online via the Zoom platform (Zoom Video Communications, Inc.) due to the Covid-19 pandemic.

### Procedure

Ethical approval was obtained from Bournemouth University and the Health Research Authority (IRAS Number: 238751). The RCT was pre-registered with the clinicaltrials.gov Protocol Registration and Results System (ID: NCT03759795). This study also adhered to CONSORT guidelines for reporting clinical trials [46].

Randomisation was undertaken by an academic colleague with no link to the research team. An online automated number generator was employed, utilising block randomisation to ensure that the number of participants per group was as close to parity as possible at any one time. The researcher (SR) then recruited participants and assigned them to ACT condition or WLC.

Survey data were collected online using the Qualtrics XM (Bournemouth University Account) web-based survey tool. Data were collected a week after each session to allow for the use and practice of the intervention.

### Overview of statistical methods

To examine whether there were any differences in outcome measures between conditions over time, a series of a two-factor mixed within- and between-groups analyses of variance (ANOVAs) were carried out. Bonferroni corrections were applied to adjust the p-value cut-off due to multiple (*n* = 6) outcome variables (*p* =.008). Significant interaction terms were used to indicate significant differences between conditions over time. Pairwise post-hoc comparisons with Bonferroni corrections were then carried out to examine: (1) whether when split by condition, either condition showed any statistically significant improvements across timepoints; (2) whether there were any significant differences between the ACT and WLC conditions at any specific timepoint. These tests were performed for each outcome measure separately.

Analyses were initially carried out with all participants in the treatment administered condition who completed the intervention (*n* = 68). These analyses were then repeated using the modified intention to treat protocol (mITT [47]), including all participants who started treatment, regardless of whether they completed (*n* = 69). The mITT analyses are reported in Additional Files 3.

We did not perform any a priori significance testing to examine baseline differences in demographics. This was in line with justification in the literature which suggests that once randomised, checking for such differences might not be useful (e.g [48]). Our pairwise comparisons which were performed as part of our main analysis were also used to confirm that there were no baseline differences across condition for any of our variables.

Data collection was impeded by the Covid-19 pandemic which started in the latter stages of the study, particularly affecting the timing of data collection for follow-up, and potentially increasing levels of psychological distress during the intervention. We report the most conservative results here (including the 3-month follow-up timepoint).

## Results

### Participant recruitment and attrition

**Fig. 2:**
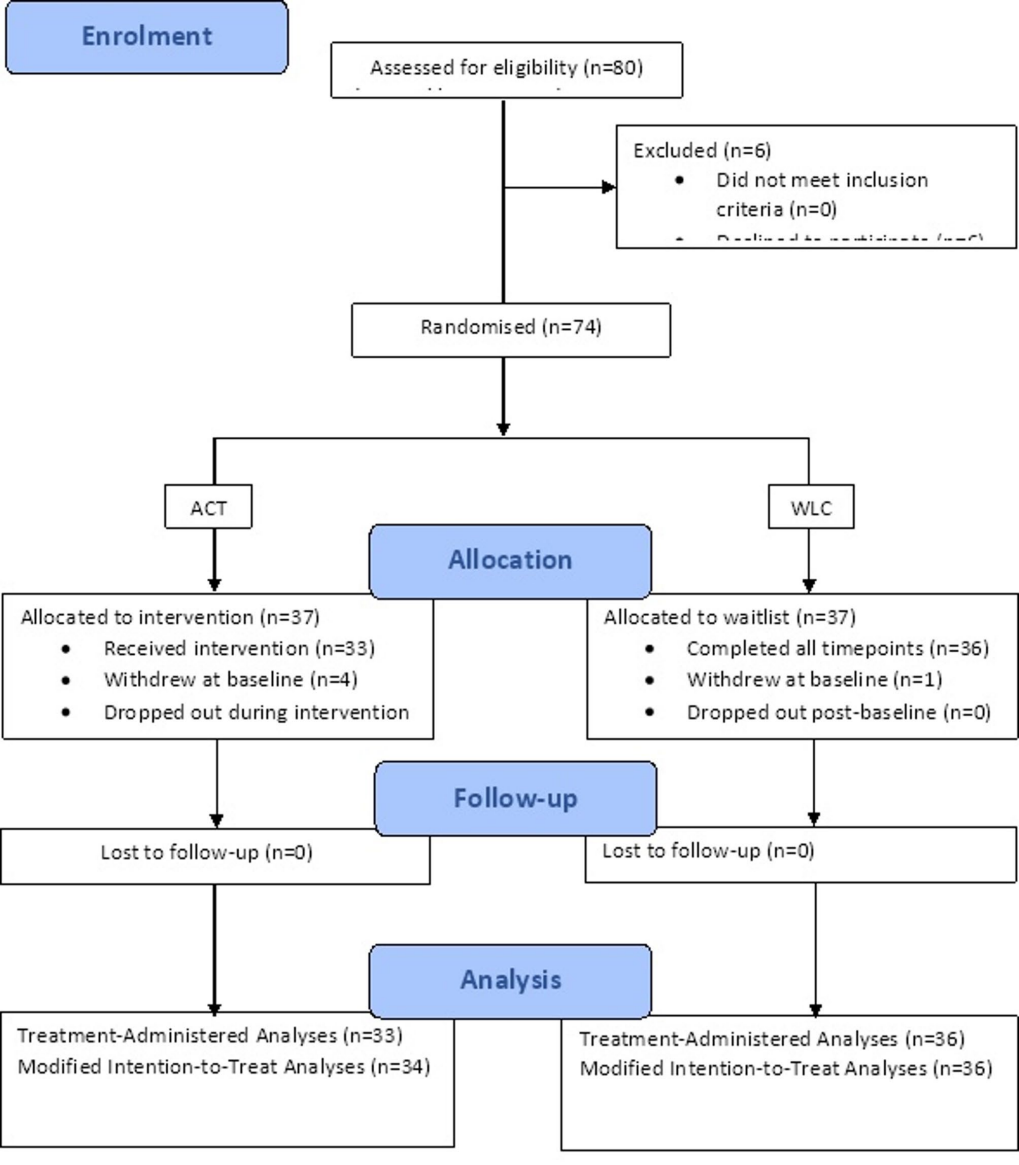
Flowchart of recruitment through the RCT for the ACT and WLC conditions

The participant flow through the trial is outlined in Fig. 2. Of the 80 participants assessed as eligible to take part, 6 declined. 37 participants were therefore randomly assigned to each condition (*n* = 74). After completing the baseline questionnaire, 4 participants withdrew from the ACT condition (before receiving any intervention) and 1 participant withdrew from the WLC condition. A further 1 participant withdrew after attending the first ACT intervention session, completing only the baseline questionnaires. The remaining participants (*n* = 68) continued the study to completion and took part in the 3-month follow-up.

### Demographic data in treatment administered analysis

Participants were aged between 26 and 50 (M = 34.93, SD = 4.85) and comprised 42 females (62%) and 26 males (38%). On average, participants had been in their current grade for 7.96 months (SD = 5.64, range 1–24). 37 participants (54.4%) worked in a University Teaching Hospital, with the remainder (*n* = 31, 45.6%) employed within a District General Hospital. Ethnicity and Speciality data are summarised in Additional Files 4.

### Assumption checks

Assumption checks were completed to assess for normal distribution of variables and equality of multiple variance-covariance matrices (see Additional Files 5). Mauchly’s W test was significant for all dependent variables, violating the assumption of sphericity; therefore, the more conservative Greenhouse-Geisser corrected p-values are given for all ANOVAs reported in our analyses.

### Treatment administered analyses (*n* = 68)

Means, standard deviations and ANOVA statistics for all measures are reported in Additional Materials 6. The ANOVA interaction effects and pairwise comparisons used to test our hypothesis (for each outcome variable), are outlined below.

#### Value Consistency (VLQC)

**Fig. 3:**
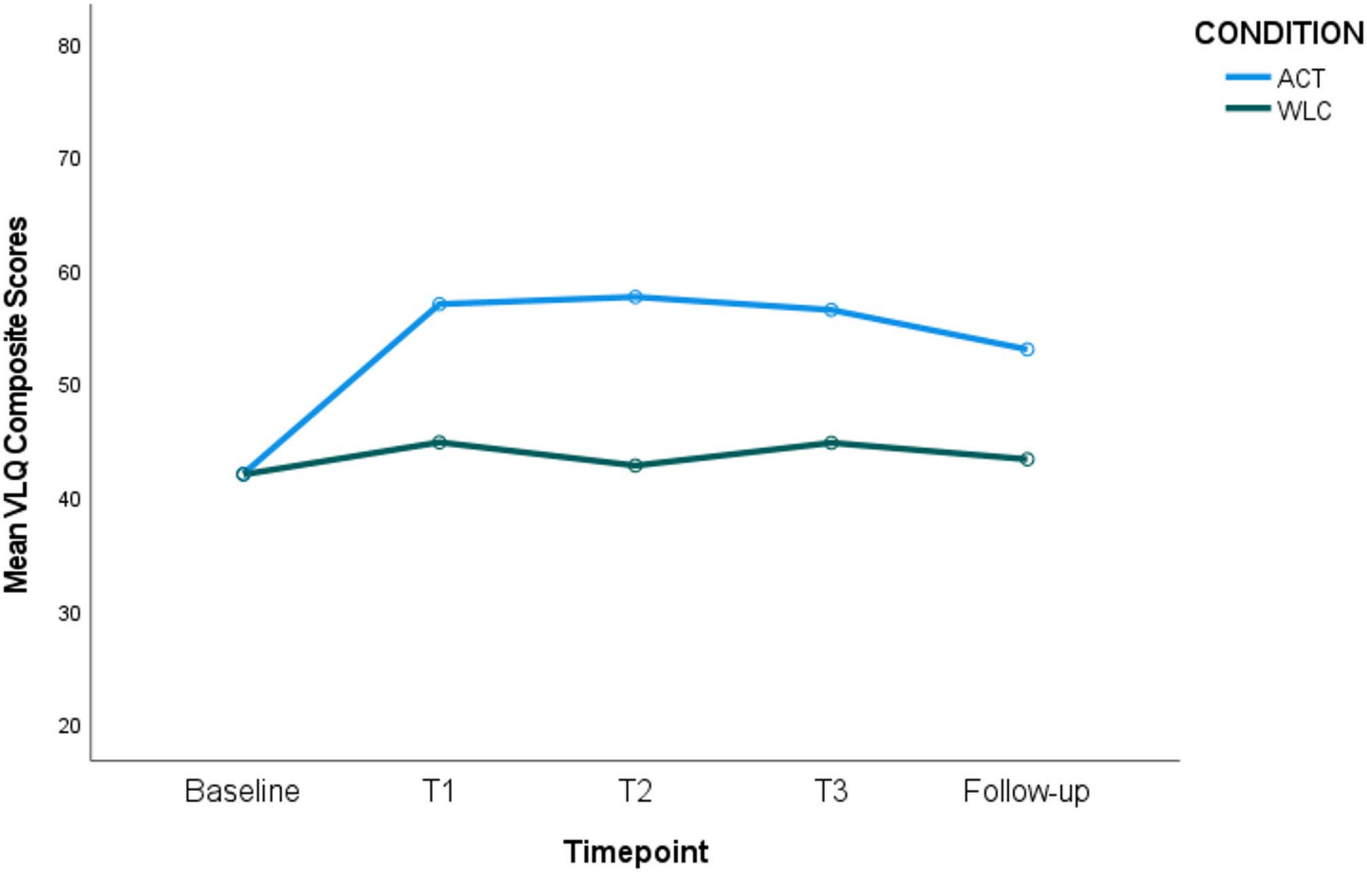
Value Consistency Scores (VLQC) for ACT versus WLC Condition over Time. Figure Note: Higher value consistency for ACT versus WLC at T1 (M_dif_ = 12.17, *p* <.001), T2 (M_dif_ = 14.85, *p* <.001) T3 (M_dif_ = 11.71, *p* <.001) and follow-up (M_dif_ = 11.71, *p* =.004). Significant improvements for ACT only from baseline > T1 (M_dif_ = 14.94, *p* <.001), baseline > T2 (M_dif_ = 15.57, *p* <.001), baseline > T3 (M_dif_ = 14.43, *p* <.001), and baseline > follow-up (M_dif_ = 10.95, *p* <.001)

The time-by-condition interaction was significant (*p* <.001, η_p_^2^ = 0.089), indicating a difference in value consistency scores for the ACT versus control conditions over time. Specifically, pairwise comparisons showed that value consistency increased significantly from Baseline to all other timepoints for the ACT condition only. When comparing conditions at each timepoint, no significant differences were observed between the ACT and control conditions at Baseline. However, value consistency scores were significantly higher for the ACT condition at Time 1, Time 2, Time 3, and Follow-up (see Fig. 3).

#### Psychological (In)flexibility (AAQ-II)

The time-by-condition interaction was not significant after applying Bonferroni corrections (*p* =.049, η_p_^2^ = 0.42), indicating no difference in psychological (in)flexibility for the ACT versus control conditions over time.

#### Work-related psychological flexibility (WAAQ)

**Fig. 4:**
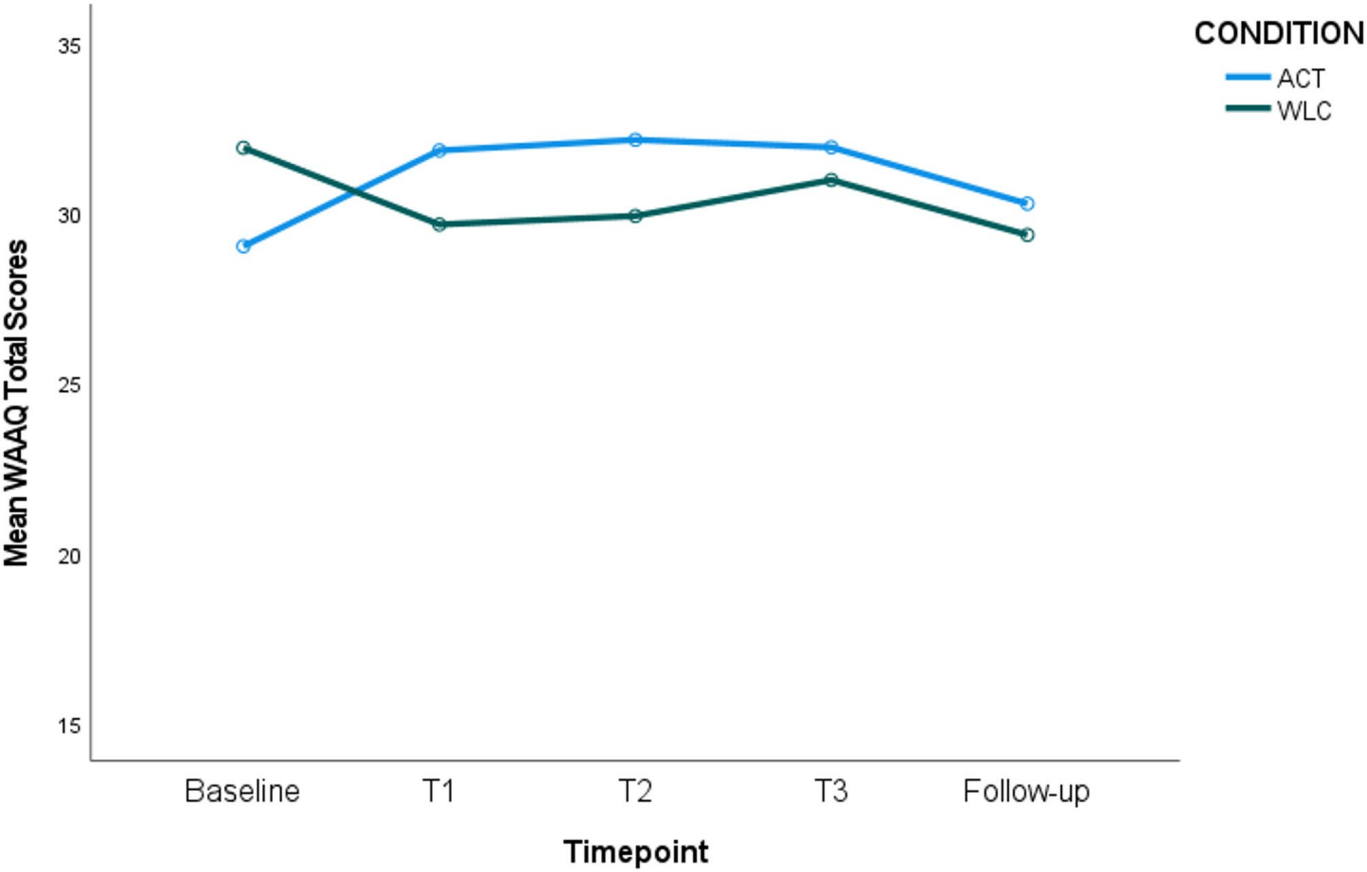
Work-Related Psychological Flexibility Scores (WAAQ) for ACT versus WLC Condition over Time. Figure Note: No significant improvements for ACT over time following Bonferroni corrections

The time-by-condition interaction effect was significant (*p* =.002, η_p_^2^ = 0.078), indicating an improvement in work-related psychological flexibility for the ACT versus control conditions over time. Specifically, pairwise comparisons showed trends in WAAQ psychological flexibility scores increasing over time for the ACT condition from Baseline to Time 1 (*p* =.019), and Baseline to Time 2 (*p* =.041), but this was not significant after applying Bonferroni corrections. There were no significant changes in psychological flexibility over time for the control condition. There were no significant differences in WAAQ scores between the ACT and control conditions at any one timepoint (see Fig. 4).

#### Brief Resilience Scale (BRS-5)

**Fig. 5:**
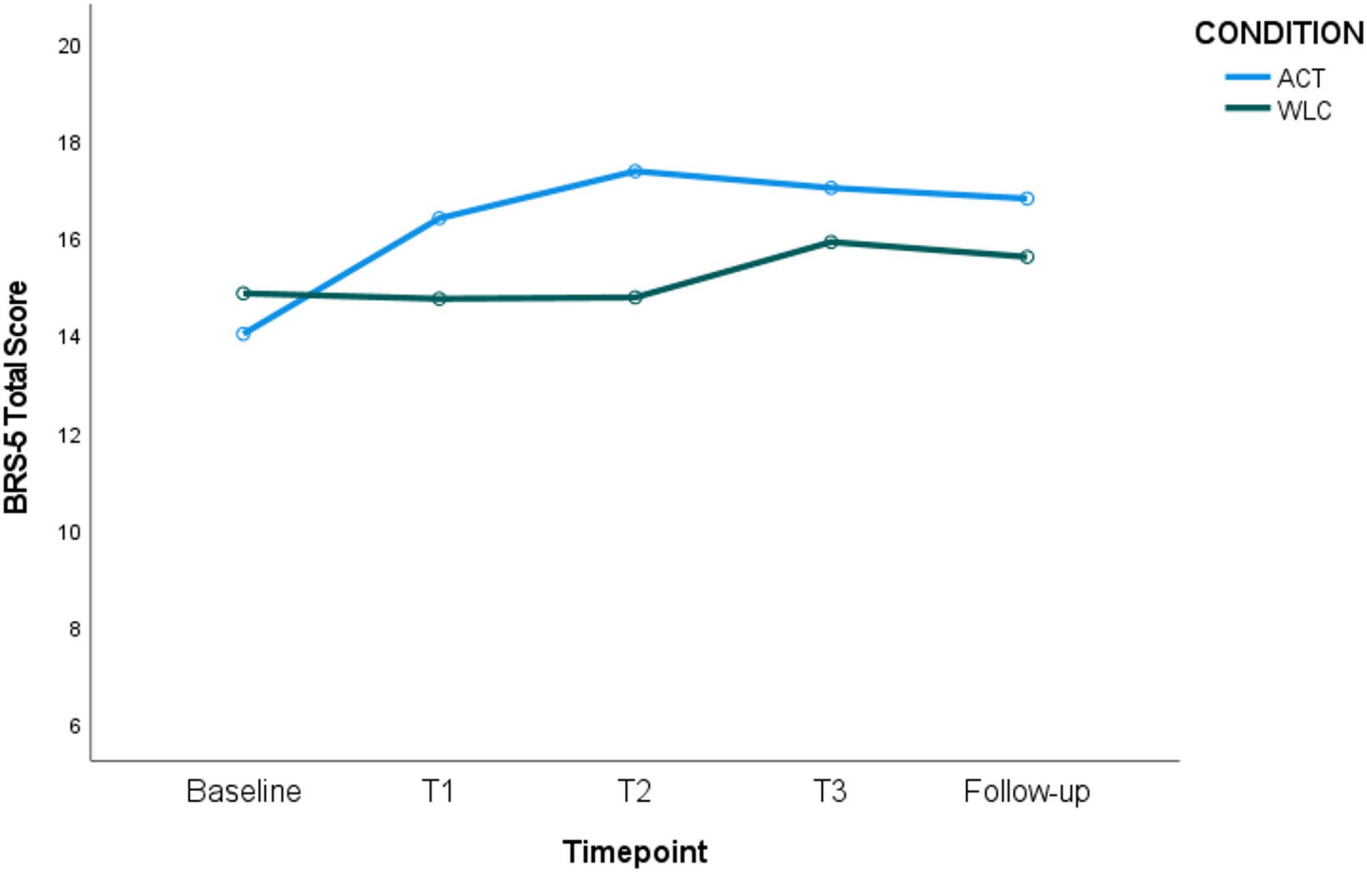
Resilience Scores (BRS-5) for ACT versus WLC Condition over Time. Figure Note: Higher resilience for ACT versus WLC at T1 (M_dif_ = 1.65, *p* =.033) and T2 (M_dif_ =2.59, *p* =.003). Significant improvements for ACT only from baseline > T1 (M_dif_ = 2.36, *p* <.001) baseline > T2 (M_dif_ = 3.34, *p* <.001), baseline > T3 (M_dif_ = 3.00, *p* <.001), baseline > follow-up (M_dif_ = 2.78, *p* <.001)

The ANOVA indicated that the time-by-condition interaction was significant (*p* <.001, η_p_^2^ = 0.08), with significant differences between resilience scores between the two conditions over time. Specifically, pairwise comparisons showed that resilience scores increased significantly between Baseline and each subsequent timepoint for the ACT condition only. No significant differences were observed between ACT and control conditions at Baseline, Time 3, or Follow-up. However, at Time 1 and at Time 2, there were significantly higher resilience scores for the ACT condition versus the control condition (see Fig. 5).

#### Self Compassion Scale (SCS)

**Fig. 6:**
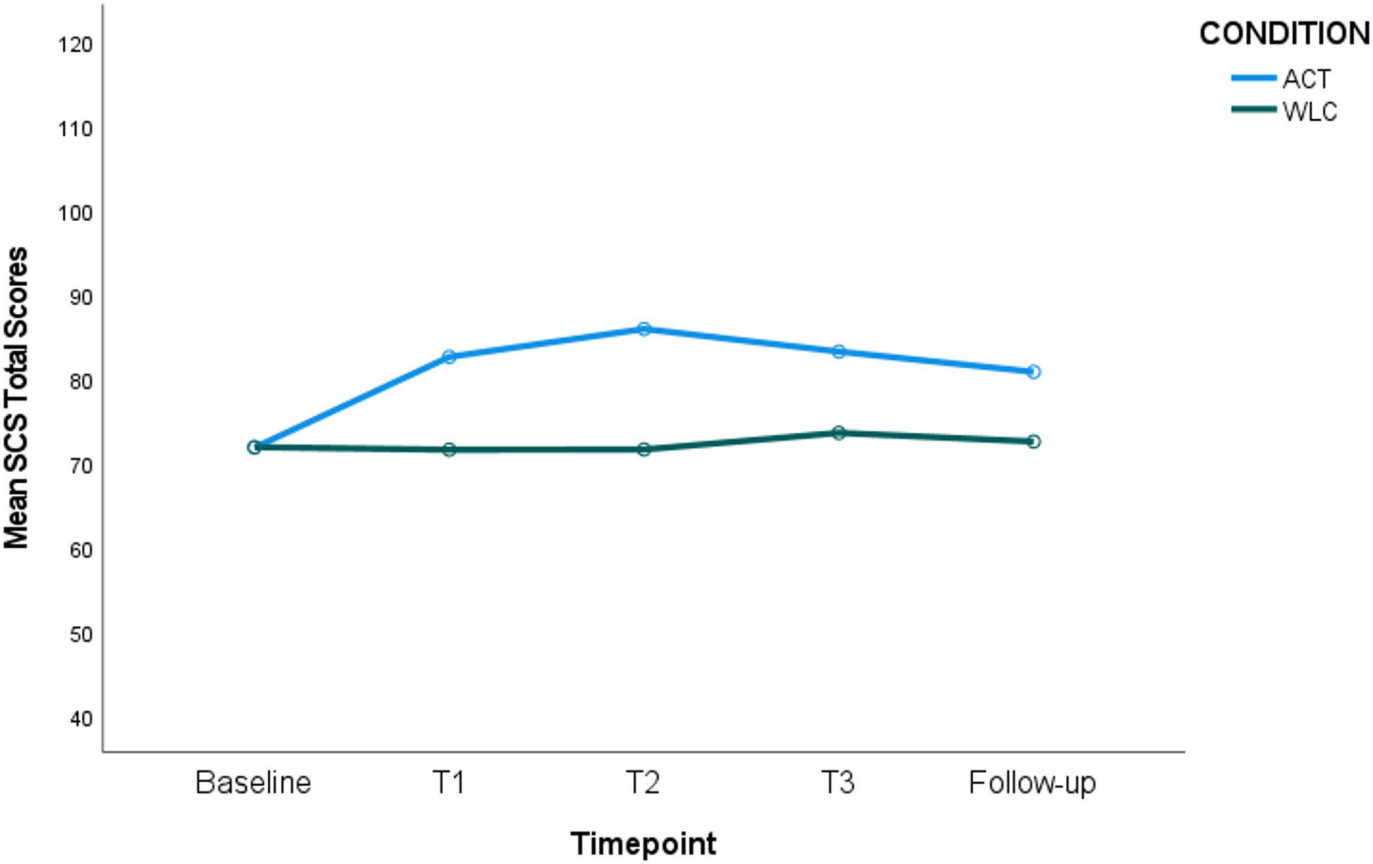
Self Compassion Scores (SCS) for ACT versus WLC Condition over Time. Figure Note: Higher self-compassion for ACT versus WLC at T1 (M_dif_ = 11.04, *p* =.003), T2 (M_dif_ =14.32, *p* <.001) T3 (M_dif_ =9.66, *p* =.012) and follow-up (M_dif_ =8.28, *p* =.022). Significant improvements for ACT only from baseline > T1 (M_dif_ = 10.78, *p* <.001), baseline > T2 (M_dif_ = 14.09, *p* <.001), baseline > T3 (M_dif_ = 11.41, *p* <.001), and baseline > follow-up (M_dif_ = 9.00, *p* <.001)

The time-by-condition interaction was significant (*p* <.001; η_p_^2^ = 0.10), indicating a difference in self-compassion scores for the ACT versus control conditions over time. Specifically, pairwise comparisons showed that there was a significant increase in self-compassion between the Baseline and all other timepoints for the ACT condition only. When comparing the two conditions at each timepoint, no significant differences were observed at Baseline, but self-compassion scores were significantly higher for the ACT condition at Time 1, Time 2, Time 3 and Follow-up (see Fig. 6).

#### Emotional Distress (DASS-21)

Across baseline to follow-up, ANOVA indicated that there was no statistically significant main effect of time or condition on Negative Emotional Distress (DASS-21) scores. The time*condition interaction effect was not significant suggesting no difference in DASS-21 scores for the ACT versus control condition over time.

### Modified intention to treat analysis

Consistent with mITT [47], analyses were repeated including all participants who started treatment (*n* = 69). The last observation carried forward approach was used to deal with missing data and all reported analyses were repeated. There were no qualitative differences in results when using the mITT dataset to those reported above. For a full description of mITT data results, see Additional Files 3.

## Discussion

### Summary of findings

This RCT investigated the effectiveness of a three session ACT-based intervention. We hypothesised that the intervention (relative to WLC) would successfully target psychological skills (psychological flexibility, values consistency) and improve wellbeing outcomes (resilience, self-compassion, and emotional distress) among trainee surgeons. As expected, trainees exposed to the intervention improved on measures of value consistency, psychological flexibility, self-compassion and resilience, compared with trainees in the WLC condition. However, some of these inter-condition differences, and improvements over time, were evident at only some timepoints of the intervention period, only partially supporting our hypothesis.

Promisingly for psychological skills, values consistency scores showed a significant improvement for the ACT condition only, with these improvements remaining at Follow-up. There were also small improvements in psychological flexibility for the ACT condition on both the WAAQ [49] and AAQ-II [37]; however, the improvements were not large enough to show a significant difference between WLC and ACT conditions at any one timepoint and were not maintained at Follow-up.

For wellbeing outcomes, self-compassion and resilience showed significant improvements over time for trainees in the ACT condition only, which remained at Follow-up. In contrast, the intervention showed no efficacy for emotional distress. Thus, our intervention demonstrated promise in fostering improvement in several wellbeing outcomes (self-compassion and resilience, but not negative emotional state) and psychological skills (values consistency, but not psychological flexibility).

### The impact of the intervention: why was our hypothesis only partially supported?

The intervention improved values consistency (an important aspect of psychological flexibility). This is consistent with previous literature, highlighting this as a key component of ACT interventions [50, 51]. It also aligns with the focus of our intervention, which prioritised values-based work. This suggests that the intervention improved participants’ ability to live life consistent with their meaningful and purposeful value domains. Such value skills have utility in improving aspects of functioning such as increased task persistence [52], decreased stress during distressing tasks [53], and improved subjective well-being [54].

The lack of sustained improvement in psychological flexibility reported by our participants is interesting, particularly as psychological flexibility is theorised as a key mechanism of therapeutic change in ACT-based work [51]. There are several possible explanations for this; although the AAQ-II [37] has historically been seen as the ‘gold standard’ for measuring psychological flexibility [55], this scale arguably neglects important components of the psychological flexibility construct as outlined in the ACT model [55]. A similar critique could be made for the work-based measure (WAAQ [49]) which focuses on ability to work rather than valued behaviour. As such, using a more comprehensive measure of psychological flexibility (e.g., the Comprehensive assessment of Acceptance and Commitment Therapy processes- CompACT [55]) may be useful in future research.

Another explanation for the lack of sustained improvement in psychological flexibility, could be the need to practice mindfulness to develop this skill [56]. While our intervention encouraged mindfulness home practices, we did not monitor adherence or engagement. More focus on developing mindfulness skills, particularly in the latter sessions, may have been needed, particularly to withstand the impact of the Covid-19 pandemic which occurred mid-intervention; the pandemic increased prevalence of anxious and depressive thoughts/emotions in healthcare practitioners [57] (and surgeons specifically [58]) potentially confounding the ability of participants to counter this level of psychological distress with their newly-acquired skills.

For well-being outcomes, while the intervention led to sustained improvements in resilience and self-compassion, it did not lead to any improvements in emotional distress. While this latter finding was unexpected, it may also be due to the confound of the pandemic onset; the DASS-21 specifically measured negative states of anxiety, stress and depression; improvements in such aspects were perhaps impeded by the systemic impact of the pandemic.

Moreover there are some modifications to the intervention which could be considered to potentially improve wellbeing outcomes. Given the lack of free time for surgical trainees, more emphasis on accessible, short mindfulness practices and forming habits around mindfulness may be particularly important [59]. Tailoring interventions to focus on barriers that surgeons may have in engaging with psychological interventions may also be useful. For example, challenging stigma around showing vulnerability/support seeking [60] in surgeons may facilitate the trainee’s capacity to reflect on difficult thoughts and emotions as part of the intervention to develop psychological flexibility skills. Focusing on ACT-based examples which draw on themes relevant to surgical trainees (such as academic demands and relationships at work [61]), may also be a helpful way to better contextualise and tailor interventions to a surgeon-specific population and thus enhance wellbeing outcomes.

Regardless of these shortcomings, particularly promising for our RCT intervention was that, despite the pandemic, improvements were seen in resilience and self-compassion. Both have been highlighted as protective factors against burnout and secondary trauma in surgeons [24], and thus particularly salient outcomes in the current healthcare climate.

### Strengths, limitations and future research

This short intervention was delivered by a non-specialist facilitator, reducing the need for extensive input from expert psychologists. While there may be an argument for improving outcomes for our intervention by employing more specialist psychology support, our research was in line with strong evidence suggesting that ACT-based interventions can be successfully delivered by non-mental health professionals [62], providing accessible and low cost support. The intervention also targeted the development of psychological skills for surgeons early in their career. There is arguably a need for such tailored interventions, given the evidence that trainees report feeling little prepared to deal with the psychological demands of their profession [10].

However, participants in this RCT were self-selecting, representing a trainee sample inevitably more motivated to engage in a psychological intervention. Moreover, there were limitations in the scales implemented; we used an abridged version of the BRS (which lacks published psychometric properties), and measures of psychological flexibility that have been criticised for their narrow scope (as outlined previously [55]). Future research needs to address such limitations. Employing qualitative measures would also be useful in understanding the clinical and experiential impact of the intervention, as well as potential mechanisms of action, in more depth.

Future research could benefit from replicating this study without the potential confound of the Covid-19 pandemic, as well as developing our understanding of the intervention’s efficacy by testing it against other active psychological treatments. Fidelity checks would also improve the veracity of this research by ensuring that the training protocol was delivered as intended. Given the lack of significant improvement in psychological flexibility and emotional distress, exploring the potential benefit of top-up training, particularly mindfulness practices, may also be useful in future research. The scalability of this type of intervention also needs to be explored. Future RCTs comparing the feasibility, acceptability and efficacy of this intervention using different formats (such as group vs. individual) will be important for understanding the widescale efficacy of such interventions.

## Conclusion

This RCT suggests that a short ACT-based intervention shows promise in facilitating sustained improvements in self-compassion, resilience, and values consistency, even within the confounding context of the Covid-19 pandemic. Future research would be useful to explore the scalability of this intervention, the potential need for more intensive or tailored mindfulness training within such trainings, as well to extend our understanding of the impact, and potential mechanisms of action of such interventions through qualitative research.

## Supplementary Information

Supplementary Material 1.

Supplementary Material 2.

Supplementary Material 3.

Supplementary Material 4.

Supplementary Material 5.

Supplementary Material 6.

## Data Availability

The datasets used and/or analysed during the current study are available from the corresponding author on reasonable request.
