# Supplementary material for: Acceptance and commitment therapy- based intervention to improve psychological skills and resilience in surgical trainees: a randomised waitlist-controlled trial
Source: BMC Surg. 2025 Jul 28;25:315. doi: 10.1186/s12893-025-03059-5 (PMC12302558; doi:10.1186/s12893-025-03059-5)
Supplement: Supplementary file 1 — Supplementary Material 1. [file 12893_2025_3059_MOESM1_ESM.docx]

**Additional Materials 1: Cronbach Alphas (Scale Internal Consistency) for our sample reported at each Timepoint for each variable.**

Table S1

*Internal Consistency of each scale across timepoint among our sample*

| Scale | Baseline (α) | Time 1 (α) | Time 2 (α) | Time 3 (α) | Follow-up (α) |
| --- | --- | --- | --- | --- | --- |
| BRS | .77 | .81 | .87 | .88 | .86 |
| SCS | .91 | .93 | .94 | .94 | .94 |
| AAQ | .92 | .93 | .93 | .96 | .95 |
| WAAQ | .91 | .89 | .91 | .93 | .90 |
| DASS-21 | .77 | .88 | .89 | .91 | .92 |

*Note: Cronbach alpha (α) measure of scale internal consistency reported at each time point.*
