# Supplementary material for: Acceptance and commitment therapy- based intervention to improve psychological skills and resilience in surgical trainees: a randomised waitlist-controlled trial
Source: BMC Surg. 2025 Jul 28;25:315. doi: 10.1186/s12893-025-03059-5 (PMC12302558; doi:10.1186/s12893-025-03059-5)
Supplement: Supplementary file 2 — Supplementary Material 2. [file 12893_2025_3059_MOESM2_ESM.docx]

**Additional Materials 2: Table summarising the ACT Protocol used (based on Flaxman et al (38)).**

| Session 1- Introduction to ACT-Based Training | |
| --- | --- |
| Welcome, Introduction and Two skills Diagram | The participant was asked to carry out an exercise to reflect on attentional focus (discuss activities done in the present moment, and those done on ‘automatic pilot’.) A diagram was used to introduce the interrelated concepts of mindfulness and value-based action. |
| Introduction to Mindfulness | A practical mindfulness ‘Raisin Exercise’ was used to cultivate present moment awareness to sensory experiences while eating a raisin. |
| Introduction to Value-Based Action | A value sort card task was used, to help the participant to identify personal valued directions in their life. The participant was asked to pick the top 5 values that they perceived as the most important for them. |
| Use of Metaphors | The ‘Compass Metaphor’ was used to explain the difference between values, value-based actions and goals. The ‘Two Sheets of Paper’ technique was then used to illustrate the aim of the training, i.e., accepting unhelpful thoughts and emotions, but holding these ‘more gently’ so that they are less of a barrier to taking meaningful valued action. |
| Home Practice | The participant was asked to engage mindfully in one value-based action and notice any internal barriers (such as difficult thoughts or feelings) that interfered with this action.  They were also asked to practice a 10-minute mindful breath exercise using a provided audio recording from HB (Clinical Psychologist). Participants were encouraged to practice this 2-3 times per week.  Handouts and diaries were provided to help with homework setting. |
| Session 2- Untangling from Internal Barriers to Value-Based Action | |
| Mindfulness of Breath and Body Practice | The participant was invited to join a 10-minute mindfulness of body and breath practice. |
| Home Practice Review | Participants reviewed progress and reflected on their experiences and barriers to the homework tasks. |
| Passengers on the Bus Metaphor | The ‘Passengers on the Bus’ metaphor was used to illustrate the ways in which difficult thoughts and feelings can unhelpfully guide action, and to introduce the concept of cognitive diffusion (i.e., a skill to help move in a valued direction regardless of any internal barriers) |
| Physicalizing Exercise | The guided mindfulness and visualization ‘Physicalizing Exercise’ focused on awareness and acceptance of emotional experiences, particularly in terms of physical manifestations of difficult emotions in the body. |
| Home Practice | The participant was asked to set short, medium and long-term SMART goals in the service of a value in two areas of their life (such as work or relationships). They were asked to set 2 value-based actions and 2 value-based short-term goals to complete within the next 2 weeks.  They were also asked to practice a 10-minute mindful breath exercise using a provided audio recording from HB (Clinical Psychologist). They were encouraged to practice this 2-3 times per week.  The participant was also encouraged to practice mindfulness during small routine day-to-day actions (such bringing awareness to sensations while cleaning their teeth).  Handouts and diaries were provided to help with homework setting. |
| Session 3- Consolidation of Mindfulness and Value-Based Action Skills | |
| Mindfulness of Breath and Body Practice | The participant was invited to join a 10-minute mindfulness of body and breath practice |
| Home Practice review | The participant reviewed progress and reflected on their experiences and barriers to the homework tasks. |
| Assessing Value Consistency | A values consistency survey was used to help the participant to reflect on how important they thought particular values were in their life, and how effectively they had been in pursuing the important values over the past few weeks. |
| Thoughts on a Cloud Exercise | The ‘Thoughts on a Cloud’ Mindfulness Exercise was used to help the participant to practice diffusion, i.e. holding onto thoughts ‘more gently’. |
| Value-based Goal Setting | The participant was asked to choose two areas of life (different from those used in previous sessions) and to set short-term, medium term and long-term SMART goals in the service of a chosen value. |
| Home Practice | Tips for future practice were given and time for informal discussion and reflections on the sessions was given. Handouts on continuation of the ACT-based work were provided. |
