# Supplementary material for: Acceptance and commitment therapy- based intervention to improve psychological skills and resilience in surgical trainees: a randomised waitlist-controlled trial
Source: BMC Surg. 2025 Jul 28;25:315. doi: 10.1186/s12893-025-03059-5 (PMC12302558; doi:10.1186/s12893-025-03059-5)
Supplement: Supplementary file 3 — Supplementary Material 3. [file 12893_2025_3059_MOESM3_ESM.docx]

**Additional Materials 3: ANOVA output for the Modified Intention to Treat Analysis**

Table S2
Means, standard deviations and two-factor mixed ANOVA results

| Variable | Baseline | | Time 1 | | Time 2 | | Time 3 | | Follow-up | |  | ANOVA | | | | |  |
| --- | --- | --- | --- | --- | --- | --- | --- | --- | --- | --- | --- | --- | --- | --- | --- | --- | --- |
|  | M | SD | M | SD | M | SD | M | SD | M | SD | Effect | | *F* ratio | df | *p* | η_p_^2^ | |
| Resilience (BRS-5) |  |  |  |  |  |  |  |  |  |  |  | |  |  |  |  | |
| ACT | 14.15 | 3.33 | 16.45 | 3.23 | 17.39 | 3.57 | 17.06 | 3.33 | 16.85 | 3.07 | C | | 3.48 | 1,67 | .067 | .049 | |
| WLC | 14.83 | 3.15 | 14.72 | 3.00 | 14.75 | 3.20 | 15.89 | 3.97 | 15.58 | 3.62 | T | | 8.78 | 3.2, 213.6 | <.001* | .116 | |
|  |  |  |  |  |  |  |  |  |  |  | TxC | | 5.30 | 3.2, 213.6 | <.001* | .073 | |
| Self Compassion (SCS) |  |  |  |  |  |  |  |  |  |  |  | |  |  |  |  | |
| ACT | 72.18 | 14.02 | 82.64 | 14.53 | 85.85 | 16.30 | 83.24 | 15.18 | 80.91 | 14.90 | C | | 8.12 | 1,67 | .006* | .108 | |
| WLC | 71.83 | 14.14 | 71.53 | 14.46 | 71.56 | 13.55 | 73.53 | 15.22 | 72.50 | 13.93 | T | | 7.90 | 3.5, 233.9 | <.001* | .105 | |
|  |  |  |  |  |  |  |  |  |  |  | TxC | | 7.38 | 3.5, 233.9 | <.001* | .099 | |
| Psych. Inflexibility (AAQ-II) |  |  |  |  |  |  |  |  |  |  |  | |  |  |  |  | |
| ACT | 23.21 | 8.28 | 20.64 | 7.85 | 22.15 | 7.97 | 22.39 | 7.48 | 23.55 | 7.12 | C | | 1.60 | 1, 67 | .210 | .023 | |
| WLC | 22.22 | 7.85 | 24.75 | 8.32 | 25.06 | 8.57 | 24.53 | 8.59 | 25.94 | 8.27 | T | | 2.29 | 2.4, 157.8 | .096 | .033 | |
|  |  |  |  |  |  |  |  |  |  |  | TxC | | 2.92 | 2.4,157.8 | .048 | .042 | |
| Psych. Flexibility (WAAQ) |  |  |  |  |  |  |  |  |  |  |  | |  |  |  |  | |
| ACT | 29.36 | 6.98 | 32.09 | 6.16 | 32.39 | 5.74 | 31.18 | 5.36 | 30.58 | 5.74 | C | | 0.60 | 1, 67 | .443 | .009 | |
| WLC | 31.89 | 6.26 | 29.64 | 6.37 | 29.89 | 6.57 | 30.94 | 6.76 | 29.33 | 5.78 | T | | 1.89 | 2.7, 183.9 | .138 | .027 | |
|  |  |  |  |  |  |  |  |  |  |  | TxC | | 5.53 | 2.7, 183.9 | .002* | .076 | |
| Values Composite (VLQC) |  |  |  |  |  |  |  |  |  |  |  | |  |  |  |  | |
| ACT | 42.03 | 8.92 | 56.52 | 11.95 | 57.13 | 18.32 | 56.02 | 16.05 | 52.65 | 14.46 | C | | 16.21 | 1, 67 | <.001* | .195 | |
| WLC | 41.90 | 9.95 | 44.73 | 11.91 | 42.68 | 11.50 | 44.69 | 11.59 | 43.24 | 10.40 | T | | 10.64 | 3.2, 216.1 | <.001* | .137 | |
|  |  |  |  |  |  |  |  |  |  |  | TxC | | 6.14 | 3.2, 216.1 | <.001* | .084 | |
| Negative Emotion (DASS) |  |  |  |  |  |  |  |  |  |  |  | |  |  |  |  | |
| ACT | 10.21 | 5.37 | 8.00 | 5.75 | 8.67 | 6.98 | 9.45 | 6.08 | 10.61 | 7.46 | C | | 3.57 | 1,67 | .063 | .051 | |
| WLC | 10.86 | 5.48 | 12.08 | 7.81 | 12.00 | 8.04 | 11.81 | 8.69 | 13.06 | 8.41 | T | | 1.36 | 3.3, 224.3 | .254 | .020 | |
|  |  |  |  |  |  |  |  |  |  |  | TxC | | 1.20 | 3.3, 224.3 | .311 | .018 | |

*Note:* C= Condition (ACT v WLC), T= Timepoint (Baseline to Follow-up), TxC = interaction (Condition over Time). * denotes significant p values <.0. *Effect sizes: η_p_****^2^*** *< .01 = small effect; η_p_****^2^*** *.01 to .06 = medium effect;  η_p_^2^ > .06 = large effect.*
