# Supplementary material for: Acceptance and commitment therapy- based intervention to improve psychological skills and resilience in surgical trainees: a randomised waitlist-controlled trial
Source: BMC Surg. 2025 Jul 28;25:315. doi: 10.1186/s12893-025-03059-5 (PMC12302558; doi:10.1186/s12893-025-03059-5)
Supplement: Supplementary file 4 — Supplementary Material 4. [file 12893_2025_3059_MOESM4_ESM.docx]

**Additional Materials 4: Ethnicity and Speciality Data for the Sample (n=68)**

| **Demographic** | **Sample (n=68)** | **ACT condition (n= 32)** | **WLC condition (n=36)** |
| --- | --- | --- | --- |
| **Ethnicity- count (%)** |  |  |  |
| White British/Irish | 46 (67.6) | 21 (65.6) | 25 (69.4) |
| Any other White Background | 2 (2.9) | 2 (6.3) | 0 (0) |
| Black or Black British-Caribbean | 1 (1.5) | 0 (0) | 1 (2.8) |
| Any other Black background | 1 (1.5) | 1 (3.1) | 0 (0) |
| White and Black Caribbean | 1 (1.5) | 0 (0) | 1 (2.8) |
| White and Black African | 1 (1.5) | 0 (0) | 1 (2.8) |
| Japanese | 1 (1.5) | 1 (3.1) | 0 (0) |
| Chinese | 4 (5.9) | 2 (6.3) | 2 (5.6) |
| Other ethnicity | 8 (11.8) | 5 (15.6) | 3 (8.3) |
| Prefer not to say | 3 (4.4) | 0 (0) | 3 (8.3) |
| **Specialty- count (%)** |  |  |  |
| Ophthalmology | 1 (1.5) | 0 (0) | 1 (2.8) |
| Obstetrics/Gynaecology | 11 (16.2) | 5 (15.6) | 6 (16.7) |
| Urology | 9 (13.2) | 6 (18.8) | 3 (8.3) |
| General Surgery | 29 (42.6) | 13 (40.6) | 16 (44.4) |
| Vascular | 2 (2.9) | 1 (3.1) | 1 (2.8) |
| Trauma/Orthopaedic | 6 (8.8) | 1 (3.1) | 5 (13.9) |
| Cardiothoracic | 3 (4.4) | 2 (6.3) | 1 (2.8) |
| Plastic Surgery | 2 (2.9) | 0 (0) | 2 (5.6) |
| Neurosurgery | 1 (1.5) | 1 (3.1) | 0 (0) |
| Academic Surgery | 1 (1.5) | 1 (3.1) | 2 (5.6) |
| Paediatric Surgery | 2 (2.9) | 1 (3.1) | 1 (2.8) |
| Otolaryngology | 1 (1.5) | 1 (3.1) | 0 (0) |
| **Age- mean (SD)** | 34.9 (4.9) | 35.4 (5.1) | 34.6 (4.6) |
| **Gender- count (%)** |  |  |  |
| Male | 26 (38%) | 9 (28.1) | 17 (47.2) |
| Female | 42 (62%) | 23 (71.9) | 19 (52.8) |
|  |  |  |  |
