# Supplementary material for: Acceptance and commitment therapy- based intervention to improve psychological skills and resilience in surgical trainees: a randomised waitlist-controlled trial
Source: BMC Surg. 2025 Jul 28;25:315. doi: 10.1186/s12893-025-03059-5 (PMC12302558; doi:10.1186/s12893-025-03059-5)
Supplement: Supplementary file 5 — Supplementary Material 5. [file 12893_2025_3059_MOESM5_ESM.docx]

**Additional Materials 5: Data Assumption Checks**

Kolmogorov-Smirnoff tests indicated that most variables data were not normally distributed. We adopted an absolute Z-score of 3.29 for kurtosis and skewness as a cut-off point to reject the null hypothesis of the distribution not being significantly different from normal (1) . For most variables across all timepoints, Z-scores for both skewness and kurtosis were within the acceptable range, with the exception of DASS scores which were positively skewed with most values in the very low-to-low range. There were two extreme outliers identified; removing them made no difference to the overall results, and so these were left in the dataset. Box’s M test was significant for the AAQ-II scale, BRS scale and the VLQ composite score indicating unequal covariance matrices. However, multivariate tests results are considered robust in the case of approximately equal sample sizes (Pituch & Stevens, 2016).

Reference

Kim HY. Statistical notes for clinical researchers: assessing normal distribution (2) using skewness and kurtosis. Restor Dent Endod. 2013;38(1):52-54.
