# Supplementary material for: Acceptance and commitment therapy- based intervention to improve psychological skills and resilience in surgical trainees: a randomised waitlist-controlled trial
Source: BMC Surg. 2025 Jul 28;25:315. doi: 10.1186/s12893-025-03059-5 (PMC12302558; doi:10.1186/s12893-025-03059-5)
Supplement: Supplementary file 6 — Supplementary Material 6. [file 12893_2025_3059_MOESM6_ESM.docx]

**Additional Materials 6.**

Means, standard deviations and two-factor mixed ANOVA result for Main Analyses

| Variable | Baseline | | Time 1 | | Time 2 | | Time 3 | | Follow-up | |  | ANOVA | | | | |  |
| --- | --- | --- | --- | --- | --- | --- | --- | --- | --- | --- | --- | --- | --- | --- | --- | --- | --- |
|  | M | SD | M | SD | M | SD | M | SD | M | SD | Effect | | *F* ratio | df | *p* | η_p_^2^ | |
| Resilience (BRS-5) |  |  |  |  |  |  |  |  |  |  |  | |  |  |  |  | |
| ACT | 14.00 | 3.26 | 16.38 | 3.25 | 17.34 | 3.62 | 17.00 | 3.36 | 16.78 | 3.10 | C | | 2.98 | 1,66 | .089 | .043 | |
| WLC | 14.83 | 3.15 | 14.72 | 3.00 | 14.75 | 3.20 | 15.89 | 3.97 | 15.58 | 3.42 | T | | 8.98 | 3.2, 210.6 | <.001* | .120 | |
|  |  |  |  |  |  |  |  |  |  |  | TxC | | 5.48 | 3.2, 210.6 | <.001* | .077 | |
| Self-Compassion (SCS) |  |  |  |  |  |  |  |  |  |  |  | |  |  |  |  | |
| ACT | 71.78 | 14.05 | 82.56 | 14.76 | 85.88 | 16.56 | 83.19 | 15.42 | 80.78 | 15.12 | C | | 7.65 | 1,66 | .007* | .104 | |
| WLC | 71.83 | 14.14 | 71.53 | 14.46 | 71.56 | 13.55 | 73.53 | 15.22 | 72.50 | 13.93 | T | | 8.18 | 3.5, 230.9 | <.001* | .110 | |
|  |  |  |  |  |  |  |  |  |  |  | TxC | | 7.64 | 3.5, 230.9 | <.001* | .104 | |
| Psych. Inflexibility (AAQ-II) |  |  |  |  |  |  |  |  |  |  |  | |  |  |  |  | |
| ACT | 23.13 | 8.40 | 20.47 | 7.91 | 22.03 | 8.07 | 22.28 | 7.57 | 23.47 | 7.22 | C | | 1.73 | 1, 66 | .193 | .026 | |
| WLC | 22.22 | 7.85 | 24.75 | 8.32 | 25.06 | 8.57 | 24.53 | 8.59 | 25.94 | 8.27 | T | | 2.28 | 2.4, 155.3 | .097 | .033 | |
|  |  |  |  |  |  |  |  |  |  |  | TxC | | 2.91 | 2.4,155.3 | .049 | .042 | |
| Psych. Flexibility (WAAQ) |  |  |  |  |  |  |  |  |  |  |  | |  |  |  |  | |
| ACT | 29.00 | 6.76 | 31.81 | 6.05 | 32.13 | 5.62 | 31.91 | 5.21 | 30.25 | 5.52 | C | | .291 | 1, 66 | .591 | .004 | |
| WLC | 31.89 | 6.26 | 29.64 | 6.37 | 29.89 | 6.57 | 30.94 | 6.76 | 29.33 | 5.78 | T | | 1.92 | 2.7, 181.0 | .134 | .028 | |
|  |  |  |  |  |  |  |  |  |  |  | TxC | | 5.57 | 2.7, 181.0 | .002* | .078 | |
| Values Composite (VLQC) |  |  |  |  |  |  |  |  |  |  |  | |  |  |  |  | |
| ACT | 41.96 | 9.05 | 56.90 | 11.93 | 57.53 | 18.47 | 56.39 | 16.16 | 52.92 | 14.61 | C | | 16.82 | 1, 66 | <.001* | .203 | |
| WLC | 41.90 | 9.95 | 44.73 | 11.91 | 42.68 | 11.50 | 44.69 | 11.59 | 43.24 | 10.40 | T | | 10.96 | 3.2, 213.7 | <.001* | .142 | |
|  |  |  |  |  |  |  |  |  |  |  | TxC | | 6.42 | 3.2, 213.7 | <.001* | .089 | |
| Negative Emotion (DASS) |  |  |  |  |  |  |  |  |  |  |  | |  |  |  |  | |
| ACT | 10.13 | 5.43 | 7.84 | 5.77 | 8.53 | 7.04 | 9.34 | 6.14 | 10.53 | 7.57 | C | | 3.79 | 1, 66 | .056 | .054 | |
| WLC | 10.86 | 5.48 | 12.08 | 7.81 | 12.00 | 8.04 | 11.80 | 8.69 | 13.06 | 8.41 | T | | 1.37 | 3.3, 220.8 | .250 | .020 | |
|  |  |  |  |  |  |  |  |  |  |  | TxC | | 1.21 | 3.6, 220.8 | .306 | .018 | |

Note: C= Condition (ACT v WLC), T= Timepoint (Baseline to Follow-up), TxC = interaction (Condition over Time). * Denotes significant p values <.05. Effect sizes: *η_p_^2^ < 0.01 = small effect; η_p_^2^ 0.01 to 0.06 = medium effect; η_p_^2^ > 0.06 = large effect.*
